# Peripheral fractional oxygen extraction measured with near-infrared spectroscopy in neonates—A systematic qualitative review

**DOI:** 10.3389/fped.2022.940915

**Published:** 2022-08-23

**Authors:** Christina H. Wolfsberger, Nina Hoeller, Ena Suppan, Bernhard Schwaberger, Berndt Urlesberger, Britt Nakstad, Gerhard Pichler

**Affiliations:** ^1^Division of Neonatology, Department of Pediatrics and Adolescent Medicine, Medical University of Graz, Graz, Austria; ^2^Research Unit for Neonatal Micro- and Macrocirculation, Department of Pediatrics and Adolescent Medicine, Medical University of Graz, Graz, Austria; ^3^Division of Pediatric and Adolescent Medicine, Institute of Clinical Medicine, University of Oslo, Oslo, Norway; ^4^Department of Pediatric and Adolescent Health, University of Botswana, Gaborone, Botswana

**Keywords:** peripheral fractional oxygen extraction, pFOE, pOE, peripheral muscle oxygenation, near-infrared spectroscopy, microcirculation, disturbances in microcirculation

## Abstract

**Background:**

Peripheral fractional oxygen extraction (pFOE) measured with near-infrared spectroscopy (NIRS) in combination with venous occlusion is of increasing interest in term and preterm neonates.

**Objective:**

The aim was to perform a systematic qualitative review of literature on the clinical use of pFOE in term and preterm neonates and on the changes in pFOE values over time.

**Methods:**

A systematic search of PubMed, Embase and Medline was performed using following terms: newborn, infant, neonate, preterm, term, near-infrared spectroscopy, NIRS, oximetry, spectroscopy, tissue, muscle, peripheral, arm, calf, pFOE, OE, oxygen extraction, fractional oxygen extraction, peripheral perfusion and peripheral oxygenation. Additional articles were identified by manual search of cited references. Only studies in human neonates were included.

**Results:**

Nineteen studies were identified describing pFOE measured with NIRS in combination with venous occlusion. Nine studies described pFOE measured on the forearm and calf at different time points after birth, both in stable preterm and term neonates without medical/respiratory support or any pathological findings. Nine studies described pFOE measured at different time points in sick preterm and term neonates presenting with signs of infection/inflammation, anemia, arterial hypotension, patent ductus arteriosus, asphyxia or prenatal tobacco exposure. One study described pFOE both, in neonates with and without pathological findings.

**Conclusion:**

This systematic review demonstrates that pFOE may provide additional insight into peripheral perfusion and oxygenation, as well as into disturbances of microcirculation caused by centralization in preterm and term neonates with different pathological findings.

**Systematic review registration:**

[https://www.crd.york.ac.uk/prospero/], identifier [CRD42021249235].

## Introduction

Peripheral muscle oxygenation measured with near-infrared spectroscopy (NIRS) has gained increasing interest both in experimental research and for clinical use in preterm and term neonates. It has the potential and advantage to recognize early stages of sepsis and shock due to disturbances in muscular tissue microcirculation **(1)**. An important parameter of peripheral muscle NIRS measurements is peripheral fractional oxygen extraction (pFOE). This measure is assumed to provide important additional information in regard to infection or inflammation and anemia **(2, 3)**. pFOE is measured with NIRS in combination with the venous occlusion method. The NIRS technology uses near-infrared light that propagates through tissues where it is differently absorbed by oxygenated hemoglobin (HbO_2_) and deoxygenated hemoglobin (deoxy-Hb). Relative changes in HbO_2_ and deoxy-Hb in a tissue can be calculated from changes in the attenuation of light **(4, 5)**. This calculated measure provides the tissue oxygenation index (TOI) or regional tissue oxygen saturation (rSO_2_), depending on the NIRS monitor used, and calculated by the equation HbO_2_/(HbO_2_ **+** deoxy-Hb). TOI/rSO_2_ reflect mean hemoglobin oxygenation in venous (70%), capillary (20%), and arteriolar (10%) compartments **(6, 7)**, but the relative distribution might change with different pathological conditions causing disturbances in microcirculation **(8)**.

Peripheral muscle NIRS measurements can be performed on the one hand in combination with venous or arterial occlusion, or on the other hand without occlusion. The occlusion is performed using a pneumatic cuff placed around the upper arm or thigh and a NIRS optode on the lower arm or calf. During venous occlusion the pneumatic cuff is inflated to a pressure, which is above the venous pressure and below the diastolic arterial pressure. Therefore, venous outflow is interrupted and arterial inflow to the extremity is undisturbed. Changes in HbO_2_, deoxy-Hb and total hemoglobin (Hbtot) during the venous occlusion are caused by arterial inflow and the oxygen consumption of the measured tissue. This enables the calculation of blood-flow, venous-oxygen-saturation (SvO_2_) and pFOE. During arterial occlusion the cuff is inflated to a pressure above the systolic arterial pressure, and changes of HbO_2_ and deoxy-Hb are only due to oxygen consumption. Due to better feasibility, less discomfort and higher reliability due to less influence of movement artifacts, the venous occlusion method has become the preferred method when compared to the arterial occlusion method (2, 7, 9–13). Quality criteria to increase the reproducibility of peripheral muscle NIRS measurements in combination with venous occlusion have already been published (7).

Peripheral muscle NIRS measurements are mainly performed with devices able to display information of different hemoglobin fractions [peripheral HbO_2_ (pHbO_2_) and deoxy-Hb (p-deoxy-Hb)] in short time intervals (14). By combining peripheral NIRS measurements with venous occlusion, important information about oxygenation, perfusion, tissue supply, and demand of oxygen can be obtained.

All calculations in peripheral muscle NIRS are based on ΔpHbO_2_ and Δp-deoxy-Hb during the venous occlusion: pFOE (11, 15) is calculated as a ratio of peripheral muscle oxygen consumption (pVO_2_) and peripheral muscle oxygen delivery (pDO_2_): pVO_2_/pDO_2_. Therefore, pFOE reflects the regional oxygen extraction, calculated from oxygen delivery and consumption for the measured organ. pVO_2_ is calculated out of peripheral muscle hemoglobin flow (pHbflow/min), arterial oxygen saturation (SpO_2_) and peripheral muscle mixed venous saturation (pSvO_2_) using the following equation (pHbflow/min) × 4 × (SpO_2_/100-pSvO_2_) and pDO_2_ is calculated as: (pHbflow/min) × 4 × (SpO_2_/100) (7, 16, 17).

The peripheral muscle hemoglobin flow (pHbflow/min) represents the increase in total hemoglobin (ΔpHbtot)—the sum of Δp-deoxy-Hb and ΔpHbO_2_, during venous occlusion within 1 min (7, 16, 17). pSvO_2_ is calculated as the ratio of ΔpHbO_2_ and ΔpHbtot: ΔpHbO_2_p/ΔHbtot and represents mainly the venous compartment (7).

In contrast to the pFOE, which represents the relative difference/extraction from arterial to venous compartment, the peripheral fractional tissue oxygen extraction (pFTOE) is calculated from the tissue oxygenation (widely described as TOI) and SpO_2_. The measure represents the relative difference/extraction from arterial to tissue compartment, thus including smaller venous and arterial vessels and capillaries: (SpO_2_-TOI)/SpO_2_ (18).

Peripheral muscle oxygen extraction (pOE) can be calculated out of the difference of SpO_2_-SvO_2_.

The aim of the present review was to perform a systematic qualitative review of literature on pFOE and pOE measured with NIRS in combination with the venous occlusion method in preterm and term neonates. We wanted to define normal values of stable neonates, and also evaluate the use of pFOE and pOE in clinical practice by including sick neonates or neonates with pathological findings.

## Methods

Articles were identified using the stepwise approach specified in the Preferred Reporting Items for Systematic Reviews and Meta-Analysis (PRISMA) Statement (19). This systematic review was approved and registered in PROSPERO (CRD42021249235).

### Search strategy

A systematic review was performed using the electronic databases PubMed, Embase and Ovid Medline to identify articles using a predefined algorithm (Appendix), with the search terms: newborn, infant, neonate, preterm, term, near-infrared spectroscopy, NIRS, oximetry, spectroscopy, tissue, muscle, peripheral, arm, calf, pFOE, pOE, FOE, OE, oxygen extraction, fractional oxygen extraction, tissue oxygen extraction, peripheral perfusion and peripheral oxygenation. Additional published reports were identified through a manual search of references in the retrieved original papers and review articles. No language restrictions were applied. The search was performed from January 1974 through April 2022.

We included original research of only human studies providing peripheral muscle oxygen extraction measured on the forearm or calf with NIRS and the venous occlusion method in term and preterm neonates.

### Study selection

Two authors (C.W. and G.P.) independently evaluated the articles identified following the literature review for eligibility, by assessing the title and abstract of the studies. The full texts were reviewed if uncertainty remained regarding eligibility for inclusion. Disagreements were resolved through discussion and consensus between the two authors (C.W. and G.P.), who critically appraised the full text and assessed the methodological quality of the included studies. The data were analyzed qualitatively. Data extraction included study design, patients’ characteristics, study aim, device used in the study, position of the measurement, interoptode distance (cm), venous occlusion, age at assessment and duration of measurements. Furthermore, the values described in each of the included studies were analyzed qualitatively and sorted according to the mean/median gestation of the neonate (preterm and term neonates), the mean/median time point of measurement and the position of measurement (forearm, calf). The infants described in each of the included studies were divided into two groups: (I) stable infants without medical/respiratory support and without pathological findings (control groups) (II) sick infants, infants with need for medical/respiratory support, infants with pathological findings (e.g., prenatal tobacco exposure, patent ductus arteriosus).

## Results

**FIGURE 1:**
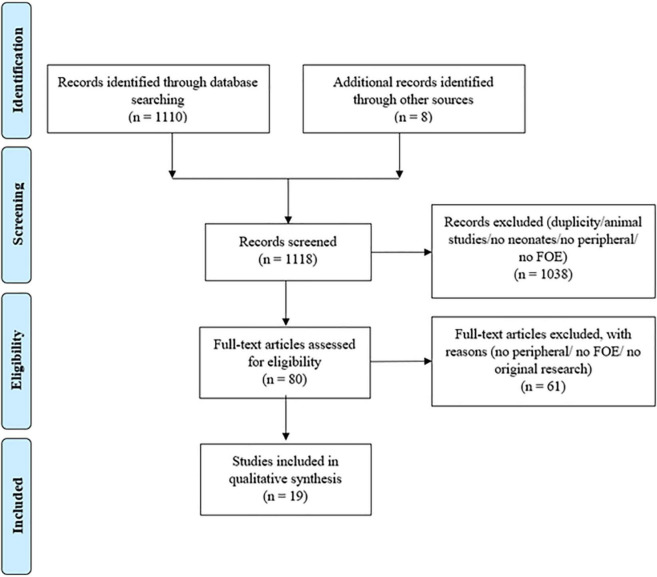
PRISMA flow chart.

Through the primary search 1,118 articles were identified; 542 articles were identified in PubMed, 352 articles in EMBASE, 216 articles in Ovid and eight articles through other sources. After removal of duplicates and exclusion: no human studies, no neonates, peripheral NIRS measurements of body parts other than extremities and missing calculation of pFOE, 80 relevant studies were assessed and 19 finally fulfilled our inclusion criteria (Figure 1). Characteristics of the included 19 studies, giving an overview of the basic data, are presented in Table 1. Reported pFOE and/or pOE values are displayed in Table 2. 16 studies described values of pFOE or pOE and three studies described pFOE (20, 21) and pOE (22) for each patient in case series that were not comparable to the other included studies or they did not provide the exact values.

**TABLE 1:** Characteristics of the included studies in preterm and term neonates, listed alphabetically according to the last name of the first author.

References	Study design	Neonates (n)	Patients	Study aim	Device	Position	Calculated value	Interoptode distance (cm)	Type of occlusion	Age at assessment	Duration of measurements
Bay-Hansen et al. (22)	Observational study	14	Preterm and term neonates	Possible relationship between peripheral and central venous saturation and co-oxymetrie	(Radiometer, Denmark	Lower leg	pOE	2.3 cm	Venous	1–17 weeks after birth	n.a.
Binder and Urlesberger (31)	Observational study	180	Preterm and term neonates	Association between leukocyte counts and peripheral tissue oxygenation	NIRO300 (Hamamatsu, Japan)	Left calf	pFOE	3.0 cm	Venous	Within the first 2 months after birth (0–1,392 h after birth)	n.a.
Bravo et al. (20)	Prospective uncontrolled case series study	16	Neonates with congenital heart defects	Effects of rescue therapy with levosimendan on cerebral and peripheral perfusion and oxygenation	NIRO300 (Hamamatsu, Japan)	Thigh	pFOE	4.0 cm	None	n.a.	7–19 h
Kissack and Weindling (13)	Observational study	24	Preterm neonates	Relationship between MABP and peripheral blood flow, pFOE in sick, ventilated babies	NIRO500 (Hamamatsu, Japan)	Right forearm	pFOE	1.0, 1.5 cm	Venous	<12 h after birth	n.a.
Mileder et al. (24)	Observational study	28	Preterm neonates	Influence of open DA on peripheral muscle oxygenation	NIRO200NX (Hamamatsu, Japan)	Lateral calf	pFOE	2.0 and 4.0 cm	Venous	1st and 3rd day after birth	n.a.
Pellicer et al. (21)	Phase I, randomized blinded study	20	Neonates undergoing cardiovascular surgery	Efficacy of milrinone and levosimendan in newborns undergoing cardiovascular surgery	NIRO300 (Hamamatsu, Japan)	Thigh	pFOE	4.0 cm	None	6–21 days after birth	During first postoperative day, 4 h at 48 and 96 h after surgery
Pichler et al. (12)	Observational study	50	Term neonates	Analyses of changes in peripheral oxygenation with postnatal age	NIRO300 (Hamamatsu, Japan)	Left forearm	pFOE	3.5 cm	Venous	Within the first week after birth	n.a.
Pichler et al. (27)	Observational study	20	Term neonates	Compare NIRS measurements on forearm and calf	NIRO300 (Hamamatsu, Japan)	Left forearm, left calf	pFOE	3.5 cm	Venous	Within the first 3 days after birth	n.a.
Pichler et al. (26)	Cohort observational study	15	Term neonates	Smoking during pregnancy and influence on peripheral tissue oxygenation	NIRO300 (Hamamatsu, Japan)	Left forearm	pFOE	3.5 cm	Venous	Within 2 days after birth	n.a.
Pichler et al. (7)	Prospective cohort observational study	40	Preterm and term neonates	To increase reproducibility, accuracy of peripheral muscle NIRS (quality criteria)	NIRO300 (Hamamatsu, Japan)	Lateral calf	pFOE, pOE	3.0 cm	Venous	13 ± 15 days after birth	Reapplication
Pichler et al. (25)	Observational cohort study	116	Preterm and term neonates	To analyze parameters potentially influencing peripheral oxygenation and perfusion	NIRO300 (Hamamatsu, Japan)	Left lateral calf	pFOE	3.0 cm	Venous	106 (2–1,392) days after birth	n.a.
Pichler et al. (3)	Observational study	66	Preterm and term neonates	Peripheral muscle oxygenation measurement in neonates with elevated CRP value	NIRO300 (Hamamatsu, Japan)	Left lateral calf	pFOE	3.0 cm	Venous	Within the 1st week after birth	n.a.
Tax et al. (32)	Observational study	38	Preterm and term neonates	Influence of perinatal asphyxia on peripheral oxygenation	NIRO300 (Hamamatsu, Japan)	Left lateral calf	pFOE	3.0 cm	Venous	<48 h after birth	n.a.
Wardle et al. (2)	Observational cohort-control study	94	Preterm neonates	Measurement of tissue oxygenation as a marker of need transfusion, normal range for forearm FOE	NIRO500 (Hamamatsu, Japan)	Upper forearm	pFOE	1.5–2.5 cm	Venous	9–37 days after birth	8 h
Wardle et al. (17)	Observational cohort-control study	30	Ventilated preterm neonates	Hypotension and influence on peripheral oxygenation	NIRO500 (Hamamatsu, Japan)	Forearm	pFOE	1.5–2.5 cm	Venous	3.5–19.0 h after birth	n.a.
Wardle (30)	Randomized controlled trial	74	Preterm neonates	Use of pFOE to guide need for blood transfusion	n.a.	Upper forearm	pFOE (calculated out of SvO_2_)	n.a.	Partial venous	3–8 days after birth	n.a.
Wolfsberger et al. (23)	Observational study	100	Preterm neonates	Peripheral muscle NIRS during the first 24 h in stable preterm neonates	NIRO200NX (Hamamatsu, Japan)	Right forearm	pFOE (VO_2_/DO_2_)	2.0 and 3.5 cm	Venous	<24 h after birth	n.a.
Zaramella et al. (28)	Observational study	43	Term neonates	Evaluate relationship between foot PI and NIRS measures	NIRO300 (Hamamatsu, Japan)	calf	pFOE	3.5 cm	Venous and arterial	1–5 days after birth	n.a.
Zaramella et al. (29)	Observational case-control study	22	Term neonates	Clamping time and affection on limb perfusion and heart hemodynamics	NIRO300 (Hamamatsu, Japan)	Calf	pFOE	n.a.	Venous	72 h after birth	n.a.

cm, centimeters; CRP, C-reactive protein; d, days; DA, ductus arteriosus; DO_2_, oxygen delivery; g, grams; h, hours; MABP, mean arterial blood pressure; n.a., not available; NIRS, near-infrared spectroscopy; OE, oxygen extraction; pFOE, peripheral fractional oxygen extraction; SvO_2_, mixed venous oxygenation; VO_2_, oxygen consumption.

**TABLE 2:** pFOE and pOE in preterm and term neonates with peripheral muscle NIRS measurements on the forearm and calf, sorted according to the mean/median time point after birth when NIRS measurements were performed.

Time point	Device	Gestational age (weeks)	References	Values	Intervention/condition
**Forearm pFOE in stable preterm neonates**					
0–6 h after birth	NIRO200	33.5 (32.5–34.1)	Wolfsberger et al. (23)	0.35 (0.29–0.40)	Stable, no intervention
7–12 h after birth	NIRO200	33.7 (33.1–34.3)	Wolfsberger et al. (23)	0.29 (0.25–0.33)	Stable, no intervention
13–18 h after birth	NIRO200	34.1 (33.2–34.6)	Wolfsberger et al. (23)	0.27 (0.23–0.29)	Stable, no intervention
19–24 h after birth	NIRO200	33.8 (32.6–34.6)	Wolfsberger et al. (23)	0.29 (0.22–0.34)	Stable, no intervention
18 (9–36) days after birth	NIRO500	29 (28–31.5)	Wardle et al. (2)	0.35 ± 0.06	None
21 (11–35) days after birth	NIRO500	26 (25–28)	Wardle et al. (2)	0.33 ± 0.05	Asymptomatic, anemic, after transfusion
**Forearm pFOE in stable term neonates**					
14.0 (0–24) h after birth	NIRO300	39 ± 1	Pichler et al. (26)	0.30 ± 0.04	Non-smoking
20.7 ± 9.6 h after birth	NIRO300	39.5 ± 1.1	Pichler et al. (12)	0.32 ± 0.13	None
25.6 (24–48) h after birth	NIRO300	39 ± 1	Pichler et al. (26)	0.35 ± 0.04	Non-smoking
38.7 ± 27.0 h after birth	NIRO300	39.5 ± 0.7	Pichler et al. (27)	0.32 ± 0.07	None
82.9 ± 20.9 h after birth	NIRO300	39.5 ± 1.1	Pichler et al. (12)	0.38 ± 0.08	None
**Calf pFOE in stable preterm neonates**					
12.5 (1.0–74.0) h after birth	NIRO200NX	34.5 ± 1.3	Mileder et al. (24)	0.3 (0.3–0.3)	Closed ductus arteriosus
106 ± 221 h after birth	NIRO300	35.5 ± 2.9	Pichler et al. (25)	0.30 ± 0.07	None
**Calf pFOE in stable term neonates**					
38.7 ± 27.0 h after birth	NIRO300	39.5 ± 0.7	Pichler et al. (27)	0.32 ± 0.07	None
41 ± 28 h after birth	NIRO300	37.5 ± 2.8	Pichler et al. (3)	0.28 ± 0.05	No CRP elevation
2.6 ± 0.9 days after birth	NIRO300	39.1 ± 1.4	Zaramella et al. (28)	0.4 ± 0.1	None
72 (61–74) h after birth	NIRO300	39 (37–42)	Zaramella et al. (29)	0.48 (0.30–0.55)	Early cord clamping time
72 (52–74) h after birth	NIRO300	40 (37–41)	Zaramella et al. (29)	0.52 (0.36–0.57)	Late cord clamping time
**Forearm pFOE in sick preterm neonates/preterm neonates with pathological findings**					
7.5 (3.5–10.3) h after birth	NIRO500	27 (27–29)	Wardle et al. (17)	0.31 (0.25–0.34)	Normotensive
8 (2–12) h after birth	NIRO500	26 (23–29)	Kissack and Weindling (13)	0.27 ± 0.06	None
16.5 (8.5–19.0) h after birth	NIRO500	27 (26–29)	Wardle et al. (17)	0.33 (0.28–0.37)	Hypotensive
12 (6–21) days after birth	n.a	29 (27–31)	Wardle (30)	0.35 ± 0.09	At transfusion
25 (13–40) days after birth	n.a	30 (27–32)	Wardle (30)	0.43 ± 0.08	At transfusion
21 (11–35) days after birth	NIRO500	26 (25–28)	Wardle et al. (2)	0.33 ± 0.05	Asymptomatic, anemic, before transfusion
23 (16–37) days after birth	NIRO500	28 (26.5–29.5)	Wardle et al. (2)	0.43 ± 0.06	Symptomatic, anemic, before transfusion
23 (16–37) days after birth	NIRO500	28 (26.5–29.5)	Wardle et al. (2)	0.37 ± 0.06	Symptomatic, anemic, after transfusion
**Forearm pFOE in sick term neonates/term neonates with pathological findings**					
14.0 (0–24) h after birth	NIRO300	39 ± 1	Pichler et al. (26)	0.37 ± 0.04	Smoking
26.0 (24–48) h after birth	NIRO300	40 ± 1	Pichler et al. (26)	0.34 ± 0.08	Smoking
**Calf pFOE in sick preterm neonates/preterm neonates with pathological findings**					
12.5 (1.0–74.0) h after birth	NIRO200NX	33.1 ± 1.3	Mileder et al. (24)	0.4 (0.3–0.4)	Open ductus arteriosus
53 (0–1,392) h after birth	NIRO300	35.5 (24.4–42.0)	Binder and Urlesberger (31)	0.29 (0.15–0.50)	None
13.0 ± 15.6 days after birth	NIRO300	35.0 ± 3.1	Pichler et al. (7)	0.37 ± 0.09	None
13.3 ± 15.7 days after birth	NIRO300	35.0 ± 3.2	Pichler et al. (7)	0.34 ± 0.07	None
**Calf pOE in sick term neonates/term neonates with pathological findings**					
106 ± 221 h after birth	NIRO300	35.5 ± 2.9	Pichler et al. (25)	26.1 ± 6.7	None
**Calf pFOE in sick term neonates/term neonates with pathological findings**					
19.0 ± 13.0 h after birth	NIRO300	38.1 ± 1.2	Tax et al. (32)	0.33 ± 0.05	Asphyxiated neonates
20.6 ± 11.7 h after birth	NIRO300	39.2 ± 1.3	Tax et al. (32)	0.28 ± 0.06	No asphyxia
41 ± 25 h after birth	NIRO300	37.7 ± 2.9	Pichler et al. (3)	0.30 ± 0.08	CRP elevation

Stable is defined as no respiratory and medical support without pathological findings/conditions. CRP, C-reactive protein; pFOE, peripheral fractional oxygen extraction; pOE, peripheral oxygen extraction.

### Peripheral fractional oxygen extraction in stable preterm and term neonates

In stable preterm neonates one study (23) described pFOE measured on the forearm 0–12 h after birth and 12–24 h after birth and one study (2) after the seventh postnatal day. Calf pFOE measurements in preterm neonates have been described in one study 12–24 h after birth (24) and in another one between day 3 and 7 (25). In stable term neonates without medical/respiratory support and/or pathological finding, pFOE measured on the forearm between 12 and 24 h after birth and 24 and 48 h after birth has been described in three studies (12, 26, 27), whereas one study measured pFOE between the third and seventh day after birth (12). Calf pFOE was measured in term neonates in two studies 24–48 h after birth (3, 27), in one study between 48 and 72 h after birth (28) and in one study between the third and seventh day after birth (29). For specification of the values see Table 3.

**TABLE 3:** pFOE in stable preterm and term neonates sorted according to the time point of measurement after birth.

Age at time of the study (mean/median)	Forearm		Calf	
		
	Preterm	Term	Preterm	Term
15 min after birth				
0–12 h after birth	0.35 (0.29–0.40)
	(Wolfsberger et al., 2020)
	0.29 (0.25–0.33)
	(Wolfsberger et al., 2020)
12–24 h after birth	0.27 (0.23–0.29)	0.30 ± 0.04	0.3 (0.3–0.3)
	(Wolfsberger et al., 2020)	(Pichler et al., 2008)	(Mileder et al., 2018)
	0.29 (0.22–0.34)	0.32 ± 0.13
	(Wolfsberger et al., 2020)	(Pichler et al., 2007a)
24–48 h after birth		0.35 ± 0.04		0.32 ± 0.07
		(Pichler et al., 2008)		(Pichler et al., 2007b)
		0.32 ± 0.07		0.28 ± 0.05
		(Pichler et al., 2007b)		(Pichler et al., 2012)
48–72 h after birth				0.4 ± 0.1
				(Zaramella et al., 2005)
>72 h–7 days after birth		0.38 ± 0.08	0.30 ± 0.07	0.48 (0.30–0.55)
		(Pichler et al., 2007a)	(Pichler et al., 2011)	(Zaramella et al., 2008)
				0.52 (0.36–0.57)
				(Zaramella et al., 2008)
>7 days after birth	0.35 ± 0.06			
	(Wardle, 1998)			
	0.33 ± 0.05			
	(Wardle, 1998)			

If different pFOE values of the same study are listed more than once in one time period, the study provided more than one pFOE value within this defined period. pFOE are display in different colors, according to the used NIRS monitor (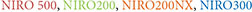). CRP, C-reactive protein; NIRS, near-infrared spectroscopy; pFOE, peripheral fractional oxygen extraction.

### Peripheral fractional oxygen extraction in preterm and term neonates with pathological findings

Concerning preterm neonates, two studies (13, 17) described pFOE measured on the forearm within 0–12 h after birth in sick, very low birth weight neonates and hypotensive neonates. One study (17) measured pFOE between 12 and 24 h in hypotensive neonates. Two studies included pFOE measurements on the forearm of preterm neonates with anemia after the seventh day after birth (2, 30). pFOE measurements on the calf were described in preterm neonates with patent ductus arteriosus between 12 and 24 h (24) and in neonates with leukocytosis between 48 and 72 h (31) after birth.

One study of term neonates of mothers who had smoked tobacco during pregnancy described pFOE measurements on the forearm 12–24 h and 24–48 h after birth (26). In one study calf pFOE measurements in different sick term neonates have been described (7). Further, two studies described calf pFOE in term neonates with asphyxia 12–24 h after birth (32) and in term neonates with elevated CRP 48–72 h after birth (3). For the specification of the values see Table 4.

**TABLE 4:** pFOE in preterm and term neonates with pathological findings/conditions sorted according to the time point of measurement after birth.

Age at time of the study (mean/median)	Forearm		Calf	
		
	Preterm	Term	Preterm	Term
15 min after birth				
0–12 h after birth	0.31 (0.25–0.34) (hypotension)
	(Wardle et al., 1999)
	0.27 ± 0.06 (sick, ventilated neonates)
	(Kissack and Weindling, 2009)
12–24 h after birth	0.33 (0.28–0.37) (hypotension)	0.37 ± 0.04 (smoking)	0.4 (0.3–0.4) (open ductus)	0.33 ± 0.05 (asphyxia)
	(Wardle et al., 1999)	(Pichler et al., 2008)	(Mileder et al., 2018)	(Tax et al., 2013)
				0.28 ± 0.06 (asphyxia)
				(Tax et al., 2013)
24–48 h after birth		0.34 ± 0.08 (smoking)		
		(Pichler et al., 2008)		
48–72 h after birth			0.29 (0.15–0.50) (elevated leukocytes)	0.30 ± 0.08 (elevated CRP)
			(Binder and Urlesberger, 2013)	(Pichler et al., 2012)
>72 h–7 days after birth				
>7 days after birth				
	0.35 ± 0.09 (anemia)			
	(Wardle, 2002)			
	0.43 ± 0.08 (anemia)			
	(Wardle, 2002)			
	0.33 ± 0.05 (anemia, transfusion)		0.37 ± 0.09 (sick neonates)	
	(Wardle et al., 1998)		(Pichler et al., 2009)	
	0.43 ± 0.06 (anemia, transfusion)		0.34 ± 0.07 (sick neonates)	
	(Wardle et al. 1998)		(Pichler et al., 2009)	
	0.37 ± 0.06 (anemia, transfusion)			
	(Wardle et al. 1998)			

If different pFOE values of the same study are listed more than once in one time period, the study provided more than one pFOE value within this defined period. pFOE are display in different colors, according to the used NIRS monitor (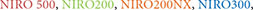, monitor not mentioned). CRP, C-reactive protein; NIRS, near-infrared spectroscopy; pFOE, peripheral fractional oxygen extraction.

### Peripheral muscle oxygen extraction in preterm and term neonates

One study defining quality criteria of peripheral muscle measurements calculated pOE (7), but not providing exact values. Further, one study (22) described pOE for each patient in case series.

## Discussion

This is the first systematic review focusing on pFOE and pOE in clinical use in stable preterm and term neonates and in sick neonates or in neonates with pathological findings, whereby we were able to identify and include 19 studies.

### Peripheral fractional oxygen extraction in stable preterm and term neonates

Peripheral fractional oxygen extraction is an important measure which provides information about oxygen extraction of the peripheral muscle on calf or forearm. pFOE has been described in 10 studies in stable preterm and term neonates without medical and/or respiratory support and without pathological findings. Therefore, this could be interpreted as normal values/reference ranges for the specified time period. However, only one study described normal values for pFOE with a sufficient sample size. Wolfsberger et al. (23) published normal values for peripheral muscle tissue oxygenation on the forearm with the NIRO200 monitor in combination with venous occlusion, in stable preterm infants within the first 24 h after birth. In this observational study they described a decrease in pFOE from 0–6 h after birth to 12–18 h after birth (23). Thereafter, a slight increase in pFOE was observed. Comparing pFOE measured within the first 6 h after birth (23) with pFOE in preterm neonates after seventh days after birth (day 9–37 after birth) (2) reveals similar values. Wolfsberger et al. (23) published values over a time period of 24 h. It may be assumed, that beside the absolute value of pFOE, changes from a baseline during a prolonged monitoring time period may provide also important information about pathological conditions.

Measurements of pFOE on the calf of preterm neonates were performed in two studies (24, 25). pFOE was similar when measured within 12–24 h (24) and between the third and seventh day after birth (25). Nevertheless, the second decimal place was not specified in one study (24), which would have provided more precise information. pFOE of calf (24) were similar when compared to forearm pFOE (23) within similar time periods.

In studies on term neonates with measurements at a specified time point or period, pFOE on the forearm (12, 26, 27) and pFOE of the calf (3, 27–29) increased from the first 12–24 h to >72 h after birth. Higher values were observed in measurements on the calf compared to the forearm. The highest pFOE values were observed in term neonates with measurements on the calf 72 h after birth (29). These studies are in accordance with the study by Pichler et al. (25) that studied preterm and term neonates. They demonstrated a significant increase of pFOE with increasing postnatal age (25). It was suggested that the changes in pFOE might be a result of changes in the muscle tone. In the latter study, in addition, a significant negative correlation between gestational age and pFOE was described (25). Other included studies do not suggest a difference in pFOE between preterm and term neonates. However, the studies report on neonates with different gestational age which could have influenced on the discrepancy in findings of gestational age impact on pFOE.

In addition to postnatal age and gestational age Pichler et al. (25) investigated potential factors influencing peripheral muscle NIRS measurements. pFOE correlated positively with birth weight, actual weight and diameter of the calf, suggesting a potential influence of the tissue composition. Furthermore, higher pFOE values correlated significantly positively with higher SpO_2_ values. Peripheral temperature and hemoglobin concentration, correlated negatively with pFOE.

#### Comparison forearm and calf peripheral fractional oxygen extraction

Forearm and calf NIRS measurements have been compared in healthy term neonates with a postnatal age of 38.7 ± 27.0 h (27). No difference in pFOE measured on forearm and on calf could be observed (27). When comparing different studies where pFOE was measured between the third and seventh day after birth, there seems to be a difference between forearm and calf. Pichler et al. described a mean pFOE of 0.38 at a mean age of 82.9 ± 20.9 h, whereas Zaramella et al. (29) described a median pFOE of 0.48–0.52 with a median age of 72 h after birth. The comparison however is difficult, since one study described mean pFOE and the other the median pFOE. Moreover, Zaramella et al. (29) did not describe the thickness of subcutaneous fat and/or the circumference of the measured limb, which might have had an influence on peripheral NIRS measurements.

### Peripheral fractional oxygen extraction in preterm and term neonates with pathological findings

In preterm neonates peripheral muscle NIRS measurements have been demonstrated to provide useful information in case of anemia (2, 30) and hypotension (13, 17), as well as in a potential influence of patent ductus arteriosus (24) and elevated leukocyte counts (31). In term neonates peripheral muscle NIRS measurements may provide additional information on exposure to certain risk factors, including maternal smoking during pregnancy (26), perinatal asphyxia (32) and elevated C reactive protein (CRP) values (3).

#### Peripheral fractional oxygen extraction and inflammation/infection

At early stages of inflammation when other routine vital parameters are still within normal ranges, pFOE has been demonstrated to provide useful information (1, 33). Binder et al. (31) examined associations between leukocyte counts and peripheral tissue oxygenation in preterm and term neonates within the first 2 months after birth. Peripheral tissue oxygen consumption decreased and vascular resistance increased with higher leukocyte counts, but no association between pFOE and leukocyte counts could be observed. Regarding CRP, Pichler et al. (3) demonstrated an impaired peripheral oxygenation and perfusion in cardio-circulatory stable preterm and term neonates with CRP elevations >10 mg/L. TOI, SvO_2_, DO_2_, and VO_2_ were significantly lower in neonates with elevated CRP levels. However, no difference in pFOE was observed in neonates with CRP >10 mg/L, compared to the control group with no CRP elevation. They assumed that a more pronounced difference could have been demonstrated if unstable cardio-circulatory neonates had been included. Furthermore, CRP elevations are seen in a variety of inflammatory conditions, which also might have influenced the results (34). Ongoing now, is a prospective trial (the pFTOE trial; ClinicalTrials.gov identifier: NCT04818762) observing a potential difference in pFTOE within the first 6 h after birth, and comparing preterm and term neonates with and without infection.

#### Peripheral fractional oxygen extraction and anemia

Blood transfusion in preterm neonates is guided by total hemoglobin and hematocrit values as well as clinical signs of anemia, even though the indications are not particularly well-defined. An observational study in preterm neonates showed that pFOE was higher in neonates with symptomatic anemia and decreased after transfusion (2). Wardle et al. (30) performed a randomized controlled trial to investigate the use of pFOE to guide need for blood transfusion in preterm neonates. For this purpose, two groups were compared where the conventional group received transfusion according to the hemoglobin value and clinical symptoms of anemia, whereby the NIRS group received transfusion when forearm pFOE was ≥0.47, or if significant clinical concerns occurred. In the NIRS group, fewer transfusions were given to the preterm neonates compared to the conventional group. However, Wardle et al. stated that pFOE failed to identify many neonates that required blood transfusion. They assumed that these results were due to the fact, that the clinicians relied on conventional indicators of transfusion or that pFOE of 0.47 as a single parameter was not sensitive enough to predict the need for transfusion.

#### Peripheral fractional oxygen extraction and arterial hypotension

Arterial hypotension is a condition that can be observed in about 20% of preterm neonates and can be associated with several neonatal morbidities (35). Early stages of shock, with signs of centralization and microcirculatory dysfunction may manifest with impaired peripheral tissue oxygenation and circulation, which might be measured by NIRS. Wardle et al. (17) observed differences in peripheral tissue oxygenation in preterm neonates with and without arterial hypotension. VO_2_ and DO_2_ were lower in hypotensive preterm neonates compared to normotensive neonates. No difference in pFOE was shown between the two groups. Another trial investigated peripheral muscle oxygenation in hypotensive preterm neonates, however, the authors only examined the TOI and not pFOE (36).

#### Peripheral fractional oxygen extraction and ductus arteriosus

Mileder et al. (24) demonstrated higher pFOE values in preterm neonates with patent ductus arteriosus compared to those with a closed ductus arteriosus. Furthermore, a significant positive correlation between pFOE and diameter of the ductus arteriosus was observed. They assumed that an increase in pFOE occurred as a result of reduced peripheral oxygen delivery, due to a steal phenomenon.

#### Peripheral fractional oxygen extraction and asphyxia

The influence of perinatal asphyxia on peripheral oxygenation and perfusion has been investigated by Tax et al. (32). NIRS parameters differ significantly between neonates with and without perinatal asphyxia, with higher pFOE values in asphyxiated neonates. Furthermore, pFOE increased with decreasing umbilical artery pH.

#### Peripheral fractional oxygen extraction and congenital heart disease

Bravo et al. (20) investigated the effect of levosimendan (reduces acute and decompensated heart failure by increasing minute volume) on hemodynamics in critically ill infants with low cardiac output. Application of seven doses of levosimendan was investigated in neonates with congenital heart defects who underwent medical or surgical cardiovascular interventions. They observed an improvement of the hemodynamic situation and a beneficial effect on cerebral and peripheral perfusion with a tendency of decreasing pFOE due to a positive balance of oxygen delivery and oxygen extraction. The positive hemodynamic effects of milrinone and levosimendan in 20 neonates undergoing cardiovascular surgery, using NIRS measurements for assessment of changes in cerebral and peripheral perfusion and oxygenation, were also observed by Pellicer et al. (21).

### Limitations

This review has some limitations. First of all, we were unable to perform a meta-analysis due to the heterogeneity in study populations, study aims, devices used and neonates’ age at assessment. Secondly, there is no clear/uniform nomenclature for peripheral oxygen extraction in the different publications. Fractional oxygen extraction (FOE), fractional tissue oxygen extraction (FTOE), tissue oxygen extraction (TOE), and oxygen extraction index (OEI) were used in different studies in part synonymously. However, there are large differences between these values. FOE describes the fractional oxygen extraction calculated out of DO_2_, VO_2_, and/or SvO_2_, all measures obtained from NIRS measurements in combination with venous occlusion. FTOE or OEI describe the fractional tissue oxygen extraction obtained without the venous occlusion method, using SpO_2_ and TOI for calculations. In contrast, TOE is calculated by using the difference of SpO_2_ and TOI without calculating a ratio to SpO_2_, like it is done for FOE and FTOE. Thirdly, different NIRS monitors were used for measurements of pFOE in different studies. The pFOE values described in the present review were mainly obtained with the different generations of the NIRO device. Hyttel-Sorensen et al. (37) showed already that the NIRO 200NX and NIRO 300, which were mainly used for pFOE measurements, differ in their absolute values, which might further influence pFOE. They described higher TOI values measured with the NIRO 200NX compared to the NIRO 300, and therefore, lower pFOE values were obtained with the NIRO 200NX. Therefore, comparison of pFOE values measured by different NIRS devices should be performed with caution and published differences should be taken into account. Fourthly, one problem of peripheral NIRS measurements is reproducibility. Recommendations to increase the validity and comparability of peripheral NIRS measurements was published in 2009 (38). Especially studies done before that publication were not performed in a standardized way, which makes the comparison difficult.

## Conclusion

Peripheral muscle NIRS measurement and especially pFOE obtained in combination with venous occlusion is a method that provides information on peripheral oxygenation and perfusion in preterm and term neonates. This review demonstrates that peripheral NIRS measurements including pFOE, both in preterm and term neonates, have the potential of providing additional information in different pathological conditions such as anemia, inflammation/infection, arterial hypotension or patent ductus arteriosus. Thus, peripheral NIRS measurements and pFOE might provide a tool of future monitoring of peripheral perfusion and oxygenation that was not routinely available until now. Furthermore, changes of pFOE from a baseline value during a prolonged monitoring, especially in conditions of cardio-circulatory failure, anemia or sepsis, might give important further information. However, an improvement in the monitoring technique, a standardized application/nomenclature and establishment of normal values for the different time points and gestational ages are needed before a routine clinical application can be introduced.

## Data availability statement

The original contributions presented in this study are included in the article/supplementary material, further inquiries can be directed to the corresponding author.

## Author contributions

CW and GP conceived the research idea and evaluated the articles. CW, GP, NH, ES, BS, BN, and BU analyzed the data, and contributed to interpretation of the results, drafting, and finalizing the manuscript. CW wrote the first draft. All authors contributed to the article and approved the submitted version.

## Appendix

Search strategies used for the systematic review
#1 newborn* OR infant* OR neonate* OR preterm* OR term*
#2 near-infrared spectroscopy* OR NIRS* OR oximetry* OR spectroscopy*
#3 tissue* OR muscle* OR peripheral* OR arm* OR calf*
#4 pFOE* OR pOE* OR FOE* OR OE* OR oxygen extraction* OR fractional oxygen extraction* OR tissue oxygen extraction* OR peripheral perfusion* OR peripheral oxygenation*
Search strategy: #1 AND #2 AND #3 AND #4
Search strategy for PubMed: last performed on 28/04/2022
